# Correction: Pereira et al. Pro-Inflammatory Cytokines Trigger the Overexpression of Tumour-Related Splice Variant RAC1B in Polarized Colorectal Cells. *Cancers* 2022, *14*, 1393

**DOI:** 10.3390/cancers17101642

**Published:** 2025-05-13

**Authors:** Joana F. S. Pereira, Cláudia Bessa, Paulo Matos, Peter Jordan

**Affiliations:** 1 Department of Human Genetics, National Institute of Health ‘Dr. Ricardo Jorge’, 1649-016 Lisbon, Portugal; joana.pereira@insa.min-saude.pt (J.F.S.P.); claudia.bessa@insa.min-saude.pt (C.B.); paulo.matos@insa.min-saude.pt (P.M.); 2 BioISI—Biosystems & Integrative Sciences Institute, Faculty of Sciences, University of Lisbon, 1749-016 Lisbon, Portugal

## Error in Figure

In the original publication [1], there was a mistake in Figure 5 as published. The lanes to show a representative RAC1B Western blot in panels 5C and 5D were inadvertently retrieved from a flipped film original containing also data from panel 5B. The corrected Figure 5, panels C and D, appears below. The authors state that the corresponding quantifying graphs in these panels were correct as published, and the scientific conclusions are unaffected. This correction was approved by the Academic Editor. The original publication has also been updated.

## Update to Supplementary Materials

Following correction of Figure 5 panels C and D, the corresponding original Western blot images were also replaced in the Supplementary Materials file, which contains all original images used in the manuscript for the assembly of representative Western blot figures.

## Figures and Tables

**Figure 5:**
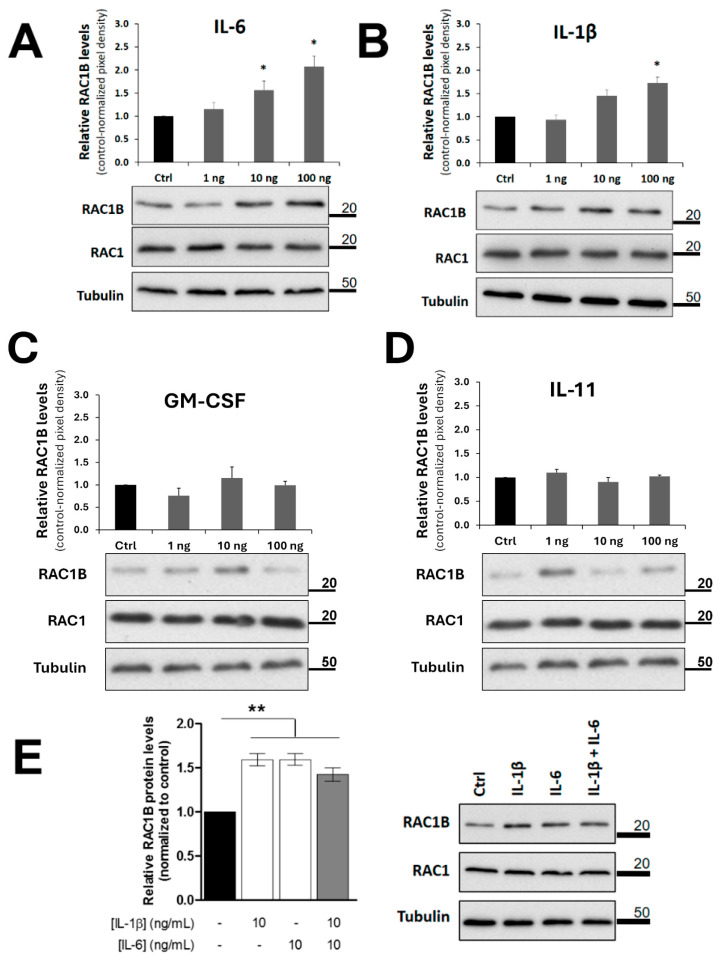
RAC1B protein levels in polarized Caco-2 cells after addition of purified candidate cytokines. Polarized Caco-2 cells were grown on filter inserts and then the indicated (**A**–**D**) purified cytokines or (**E**) cytokine combinations were added to the basolateral growth medium. Cells were lysed after 48 h, proteins analysed by SDS-PAGE and the indicated proteins detected by WB. Detection of the α-tubulin protein served as a loading control. Data are shown as fold change in RAC1B protein levels relative to control (addition of antibody solvent PBS to the medium) and represent mean values ± SEM, of at least 3 independent experiments. Statistical analysis was carried out with a one-way ANOVA test (F = 39.04, *p* < 0.001), followed by Tukey’s post hoc tests; * or ** significantly different from the corresponding control (PBS) with *p* < 0.05 or *p* < 0.01, respectively.
