# Supplementary material for: Pro-Inflammatory Cytokines Trigger the Overexpression of Tumour-Related Splice Variant RAC1B in Polarized Colorectal Cells
Source: Cancers (Basel). 2022 Mar 9;14(6):1393. doi: 10.3390/cancers14061393 (PMC8946262; doi:10.3390/cancers14061393)
Supplement: Supplementary file 1 [file cancers-14-01393-s001.zip › Suppl File S1_Original WB exposures_update.pdf]

Supplementary File S1

Original Western blot film exposures

Manuscript title:

Pro-Inflammatory Cytokines Trigger the Overexpression of Tumour-Related Splice Variant RAC1B in Polarized Colorectal Cells

Joana F. S. Pereira[1,2], Cláudia Bessa[1,2], Paulo Matos[1,2], Peter Jordan[1,2]

[1]Department of Human Genetics, National Health Institute Dr. Ricardo Jorge, Lisbon, Portugal; [2]BioISI – Biosystems & Integrative Sciences Institute, Faculty of Sciences, University of Lisbon, Lisbon, Portugal

we show box-marked in red color the areas selected from the original images that were used to assemble the final figure, also indicating the antibody used for staining

(the corresponding final Figure is shown again on the right hand of each page)

Figure 1C

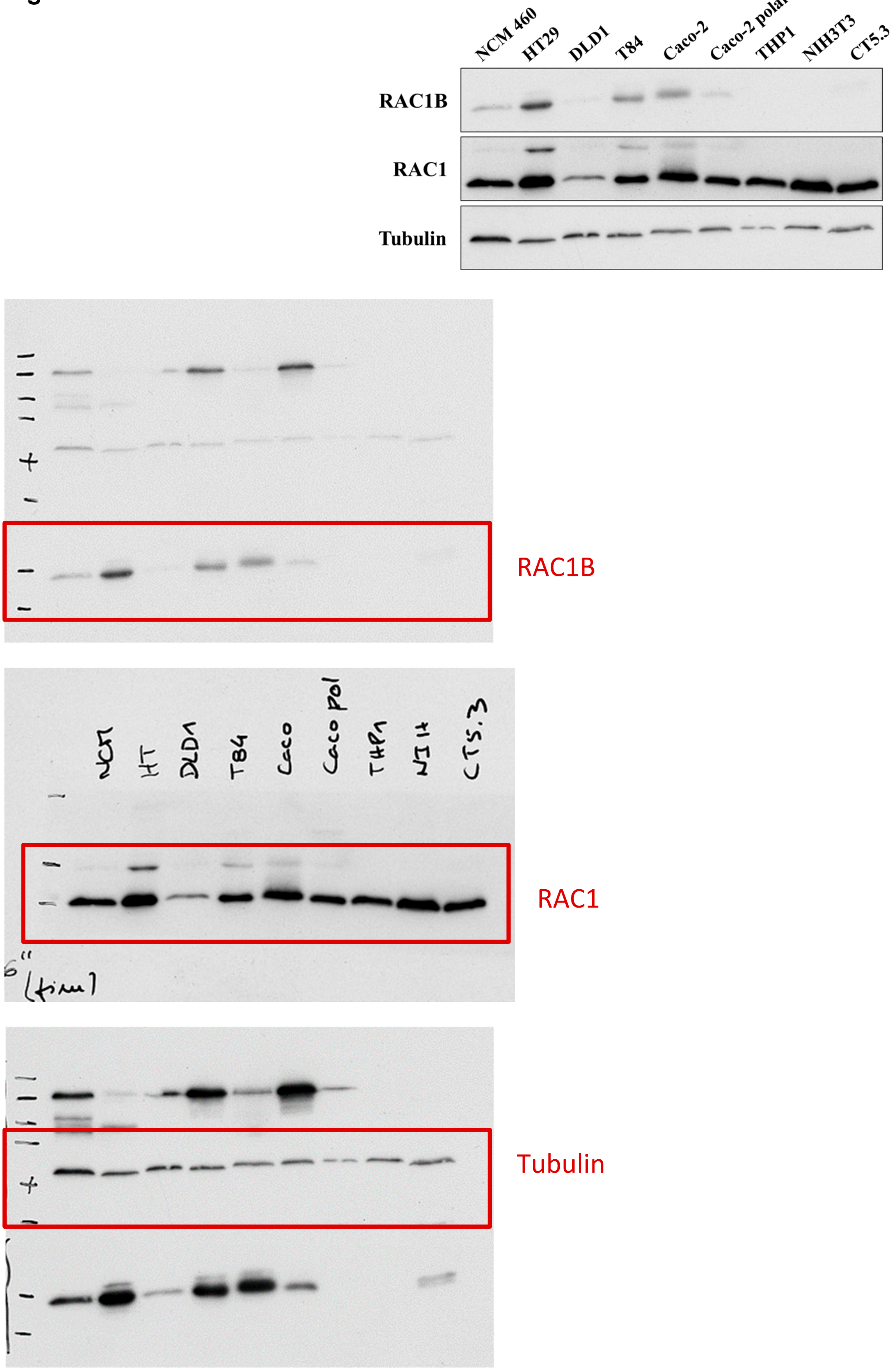

NCM 460
HT29
DLD1
T84
Caco-2
Caco-2 polar
THP1
NIH3T3
CT5.3
RAC1B
RAC1
Tubulin
RAC1B
RAC1
Tubulin

Figure 3A

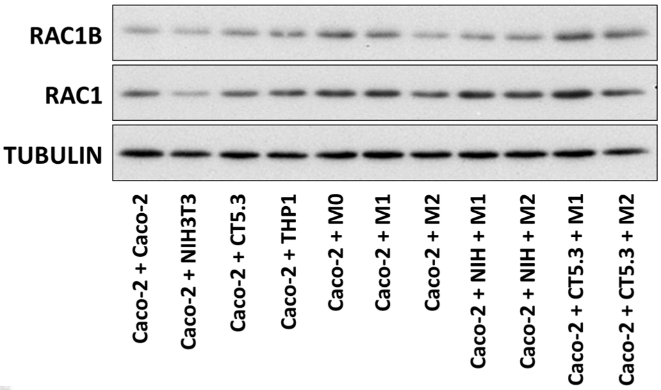
RAC1B
RAC1
TUBULIN
Caco-2 + Caco-2
Caco-2 + NIH3T3
Caco-2 + CT5.3
Caco-2 + THP1
Caco-2 + M0
Caco-2 + M1
Caco-2 + M2
Caco-2 + NIH + M1
Caco-2 + NIH + M2
Caco-2 + CT5.3 + M1
Caco-2 + CT5.3 + M2

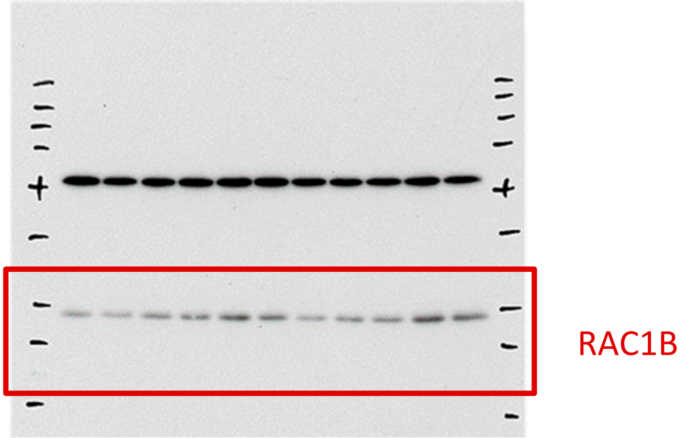
RAC1B

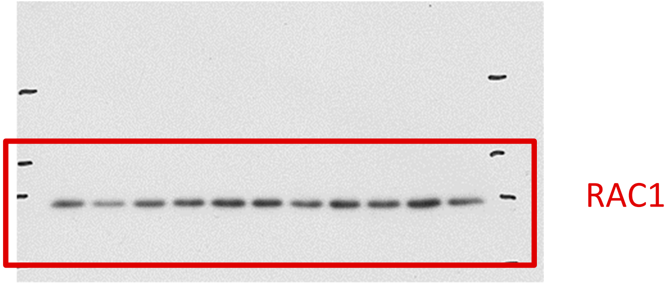
RAC1

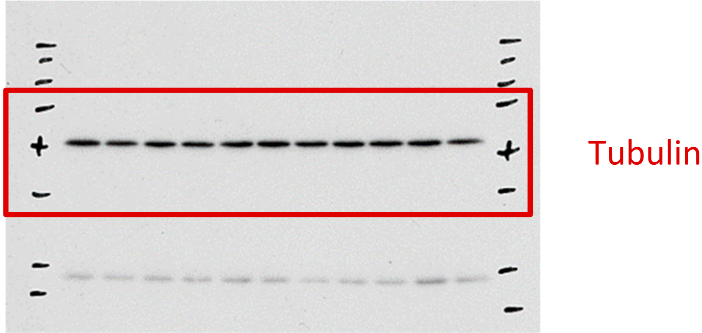
Tubulin

Figure 4A

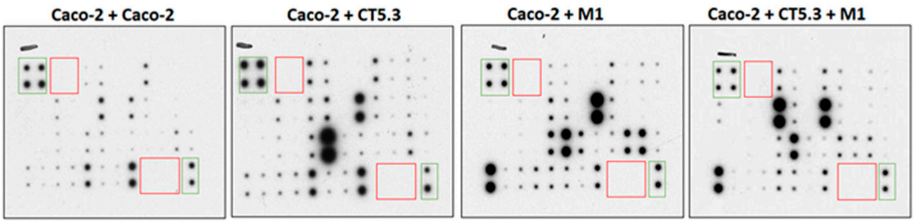

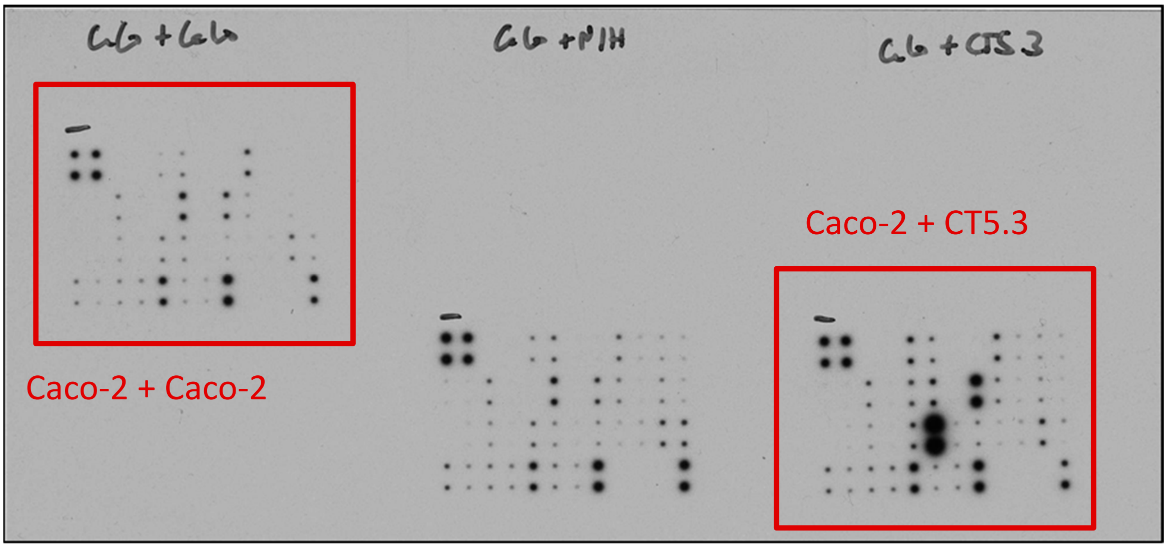

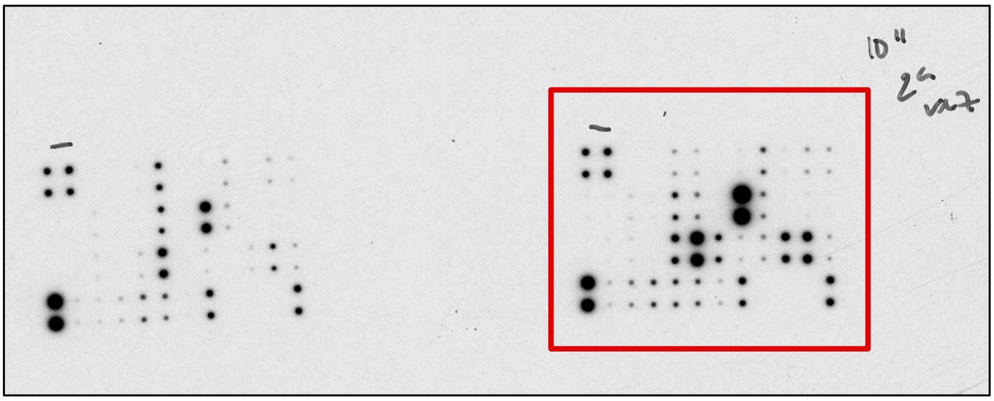

Caco-2 + M1

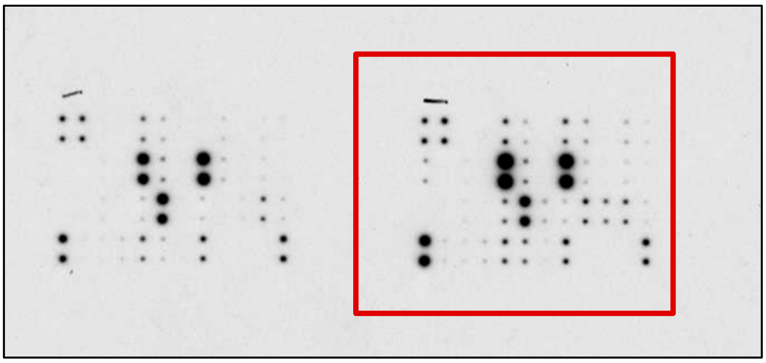

Caco-2 + CT5.3 + M1

Figure 5A

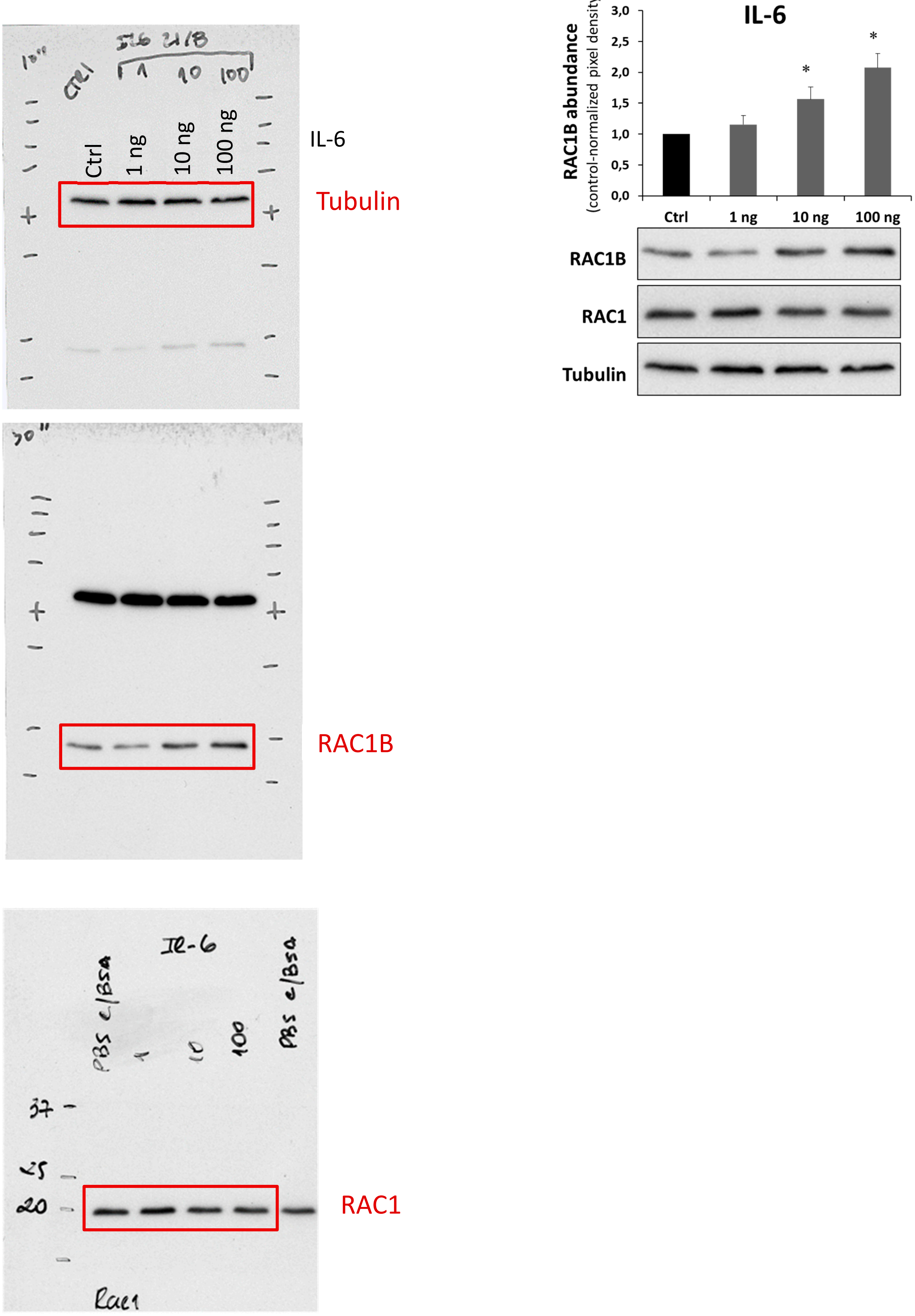
Ctrl
1 ng
10 ng
100 ng
IL-6
Tubulin
RAC1B
RAC1
IL-6
RAC1B abundance
(control-normalized pixel density)
3,0
2,5
2,0
1,5
1,0
0,5
0,0
*
*
Ctrl
1 ng
10 ng
100 ng
RAC1B
RAC1
Tubulin

Figure 5B

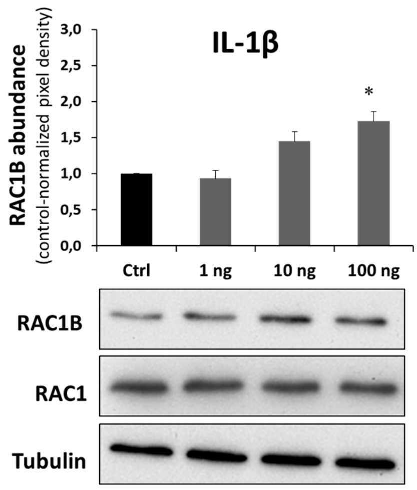

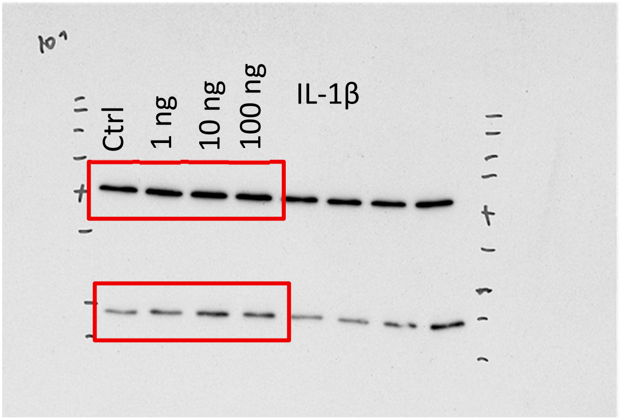

Tubulin

RAC1B

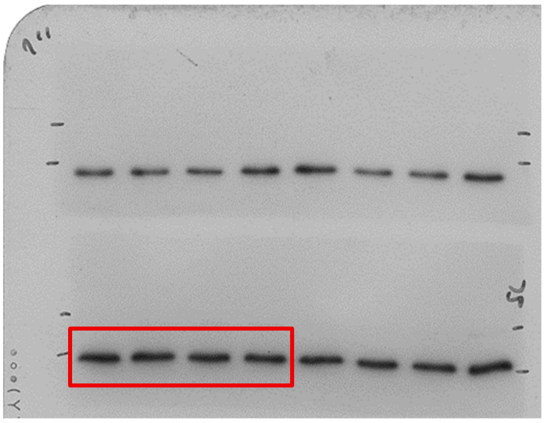

RAC1

Figures 5C + D

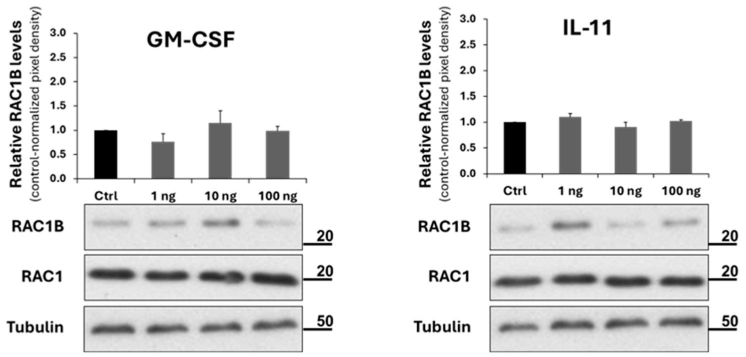
GM-CSF
IL-11
Relative RAC1B levels
(control-normalized pixel density)
3.0
2.5
2.0
1.5
1.0
0.5
0.0
Ctrl
1 ng
10 ng
100 ng
RAC1B
RAC1
Tubulin
20
20
50

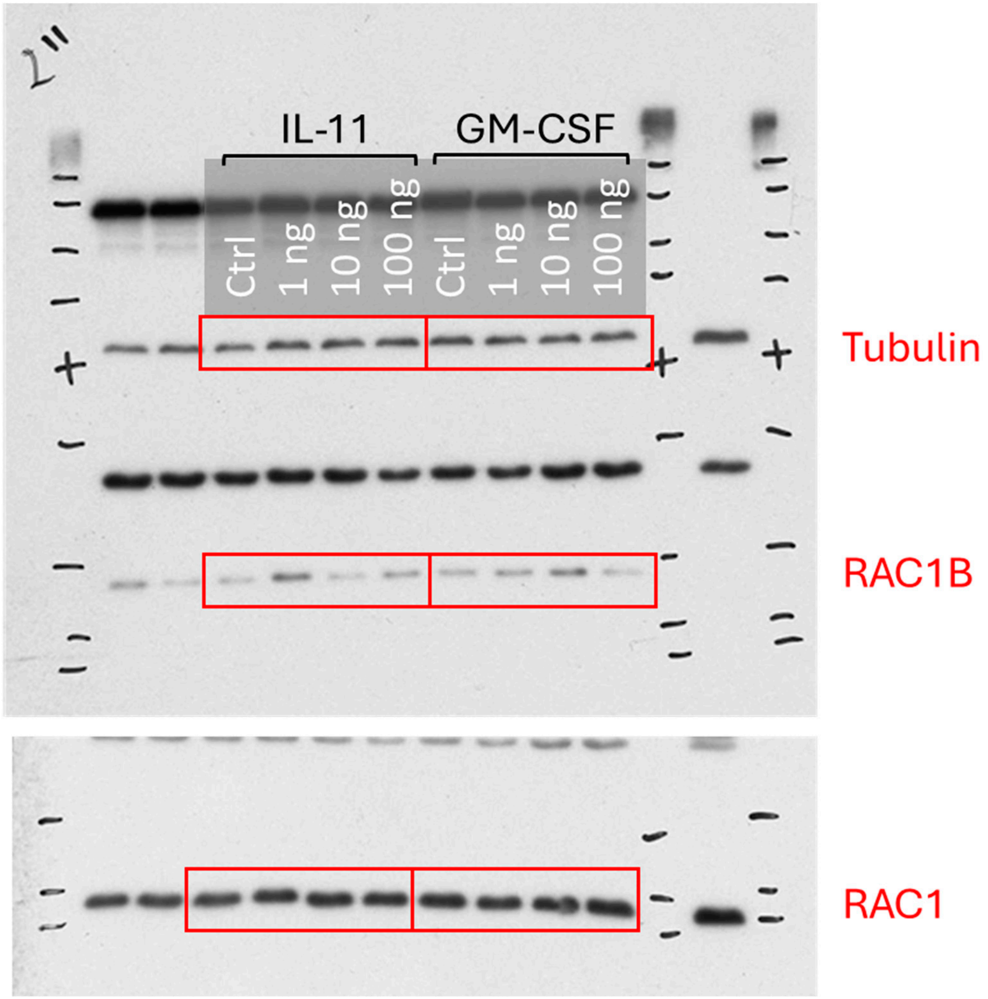
IL-11
GM-CSF
Ctrl
1 ng
10 ng
100 ng
Ctrl
1 ng
10 ng
100 ng
Tubulin
RAC1B
RAC1

Figure 6A

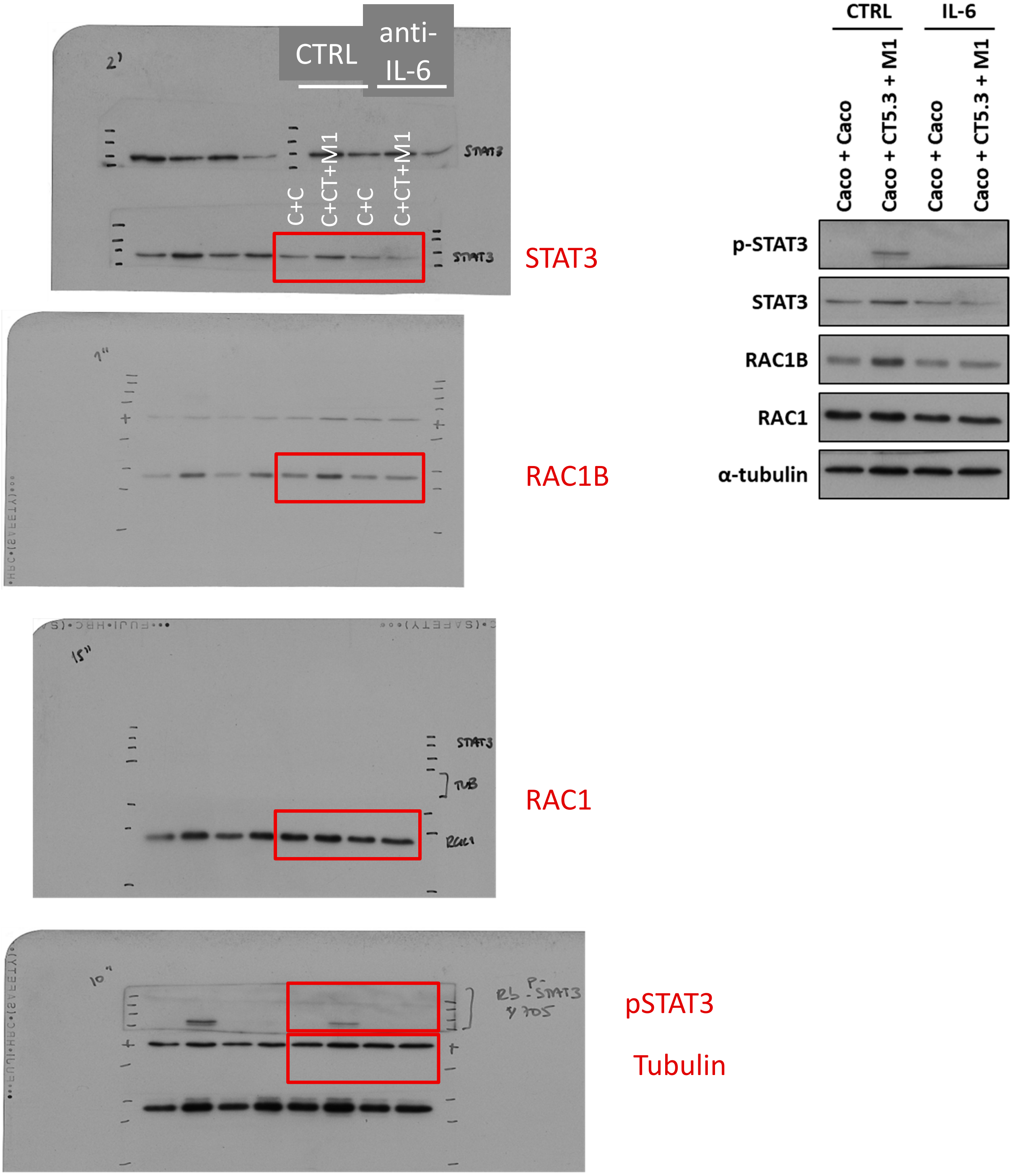
CTRL
anti-IL-6
C+C
C+CT+M1
C+C
C+CT+M1
STAT3
RAC1B
RAC1
pSTAT3
Tubulin
CTRL
anti IL-6
Caco + Caco
Caco + CT5.3 + M1
Caco + Caco
Caco + CT5.3 + M1
p-STAT3
STAT3
RAC1B
RAC1
α-tubulin

Figure 6B

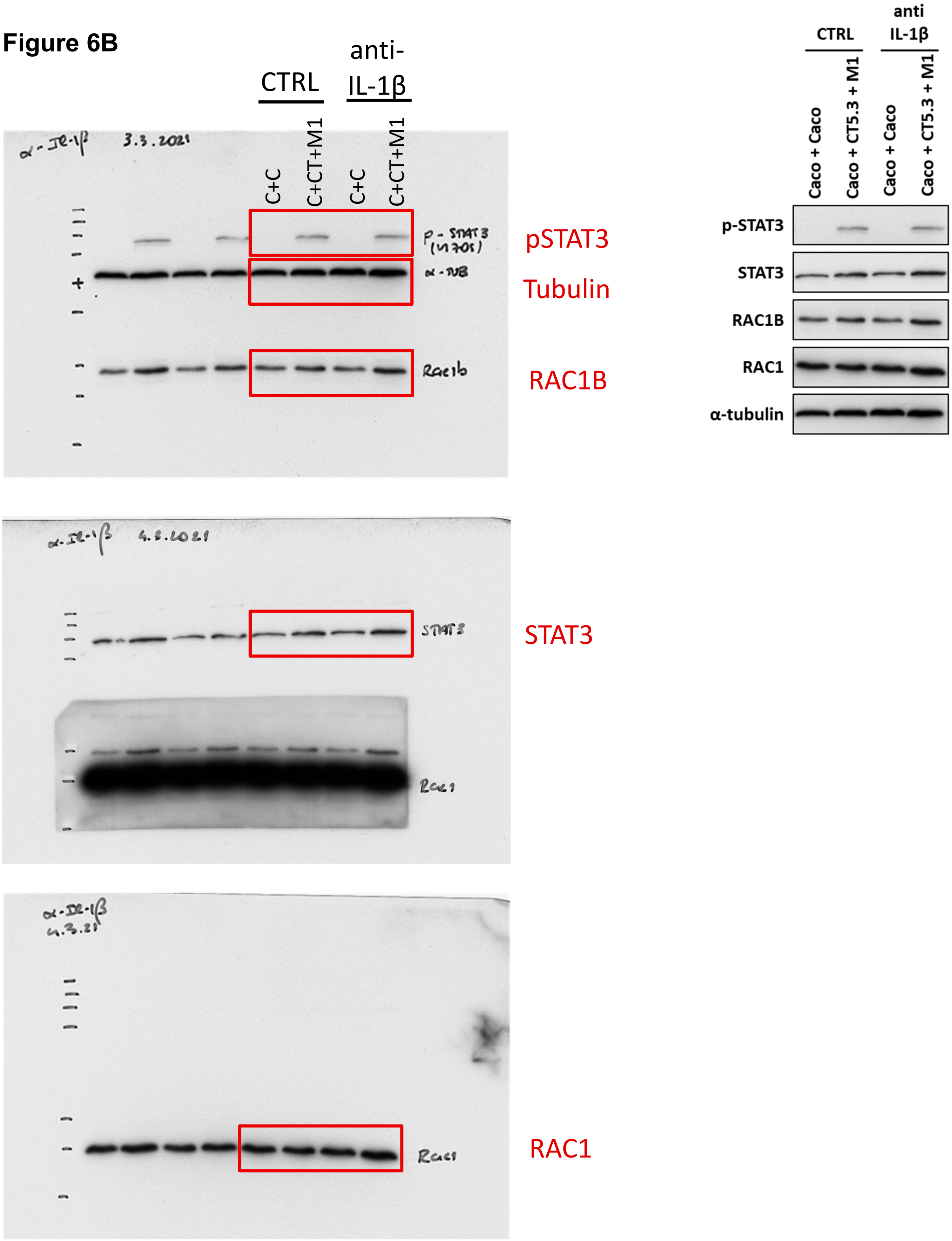
CTRL
anti-IL-1β
C+C
C+CT+M1
C+C
C+CT+M1
pSTAT3
Tubulin
RAC1B
STAT3
RAC1
CTRL
anti IL-1β
Caco + Caco
Caco + CT5.3 + M1
Caco + Caco
Caco + CT5.3 + M1
p-STAT3
STAT3
RAC1B
RAC1
α-tubulin

Figure 6C

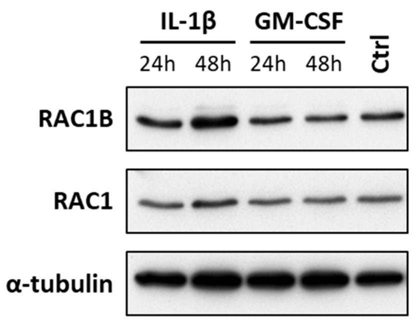
IL-1β
GM-CSF
24h 48h 24h 48h
Ctrl
RAC1B
RAC1
α-tubulin

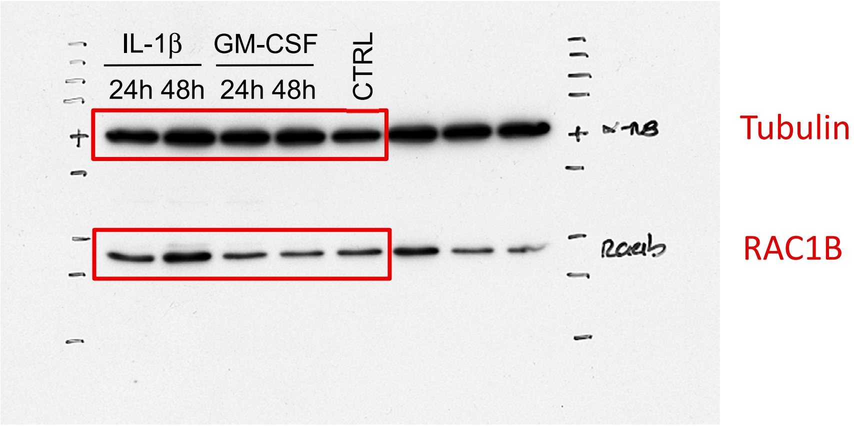
IL-1β
GM-CSF
24h 48h 24h 48h
CTRL
Tubulin
RAC1B

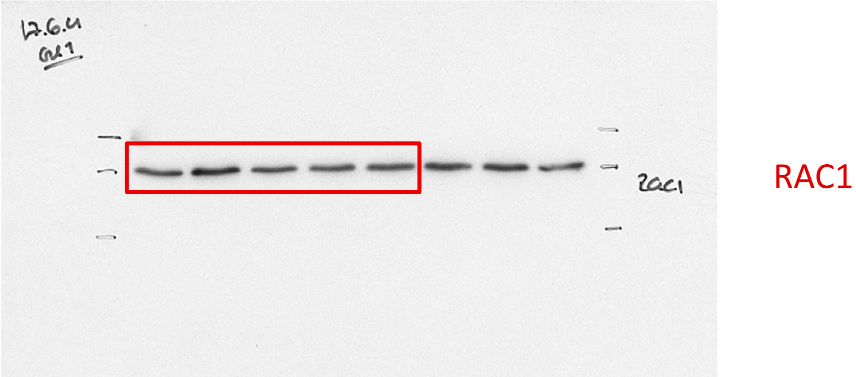
RAC1

Figure 7B

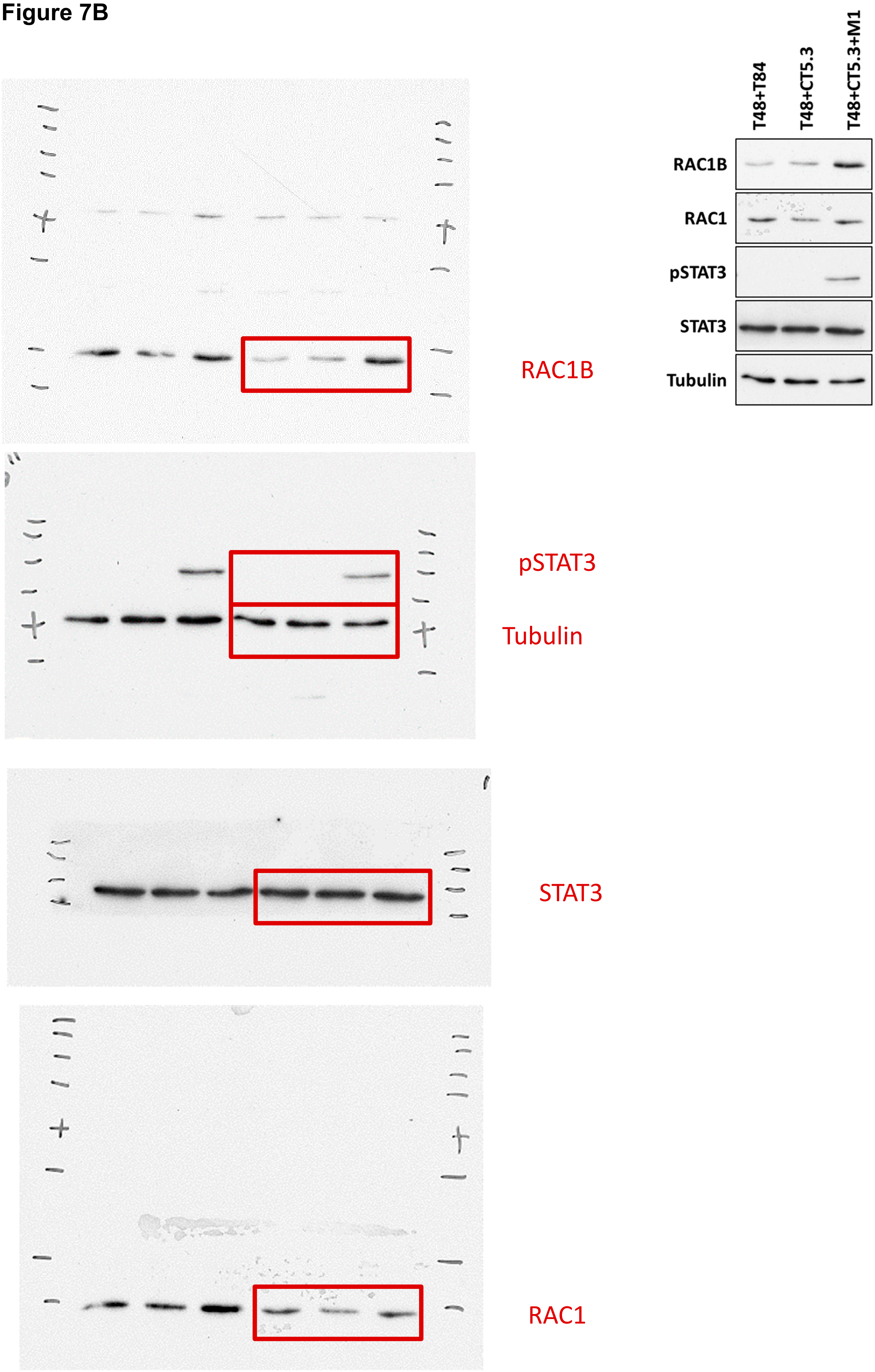
T48+T84
T48+CT5.3
T48+CT5.3+M1
RAC1B
RAC1
pSTAT3
STAT3
Tubulin
RAC1B
pSTAT3
Tubulin
STAT3
RAC1

Figure 7E

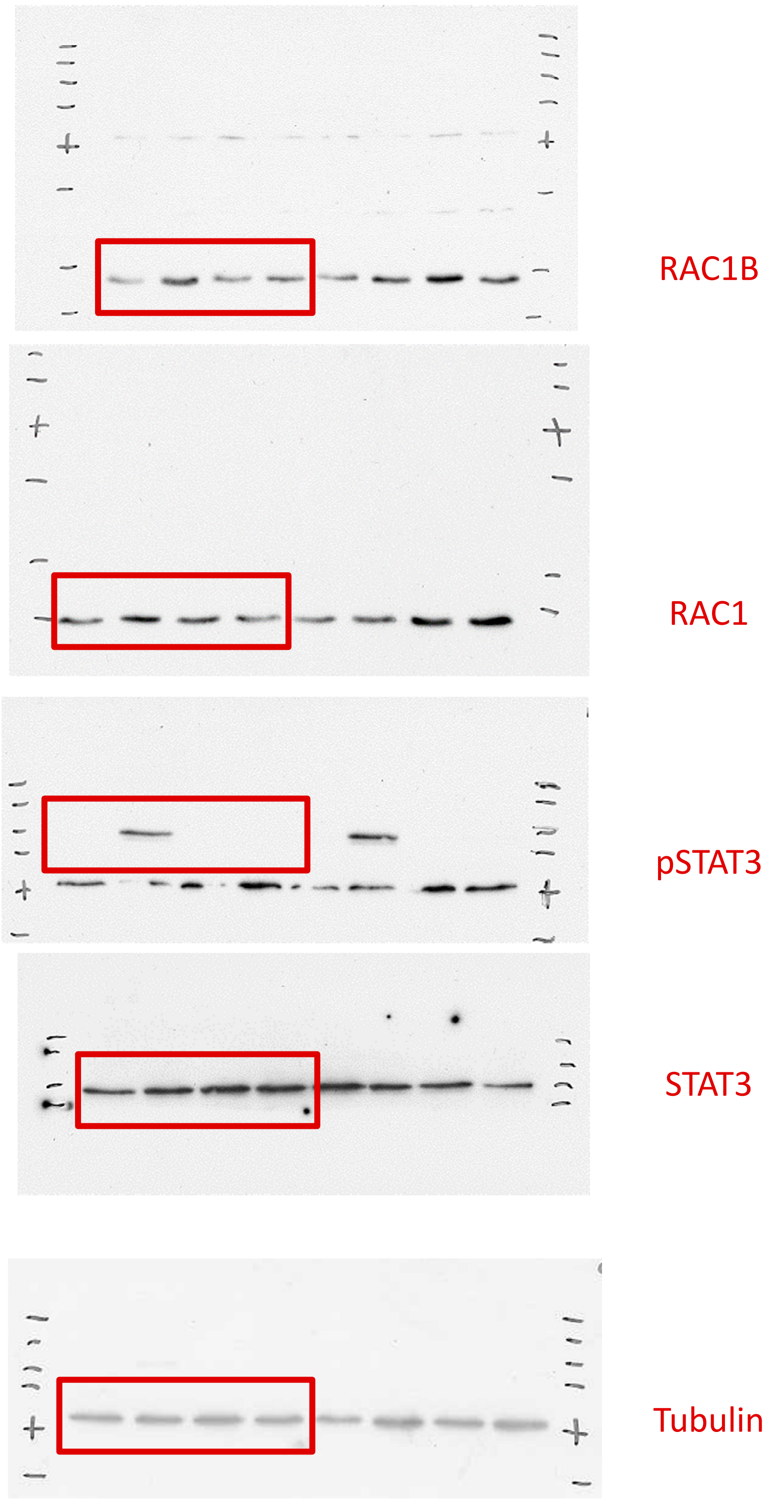
RAC1B
RAC1
pSTAT3
STAT3
Tubulin

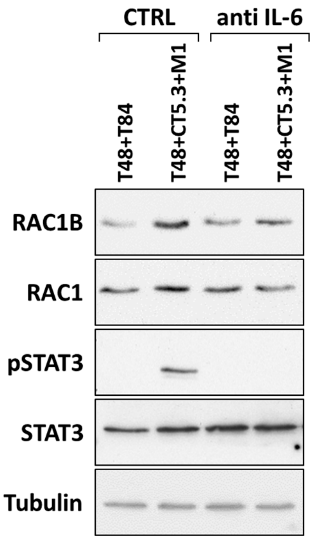
CTRL
anti IL-6
T48+T84
T48+CT5.3+M1
T48+T84
T48+CT5.3+M1
RAC1B
RAC1
pSTAT3
STAT3
Tubulin

Figure 7 G

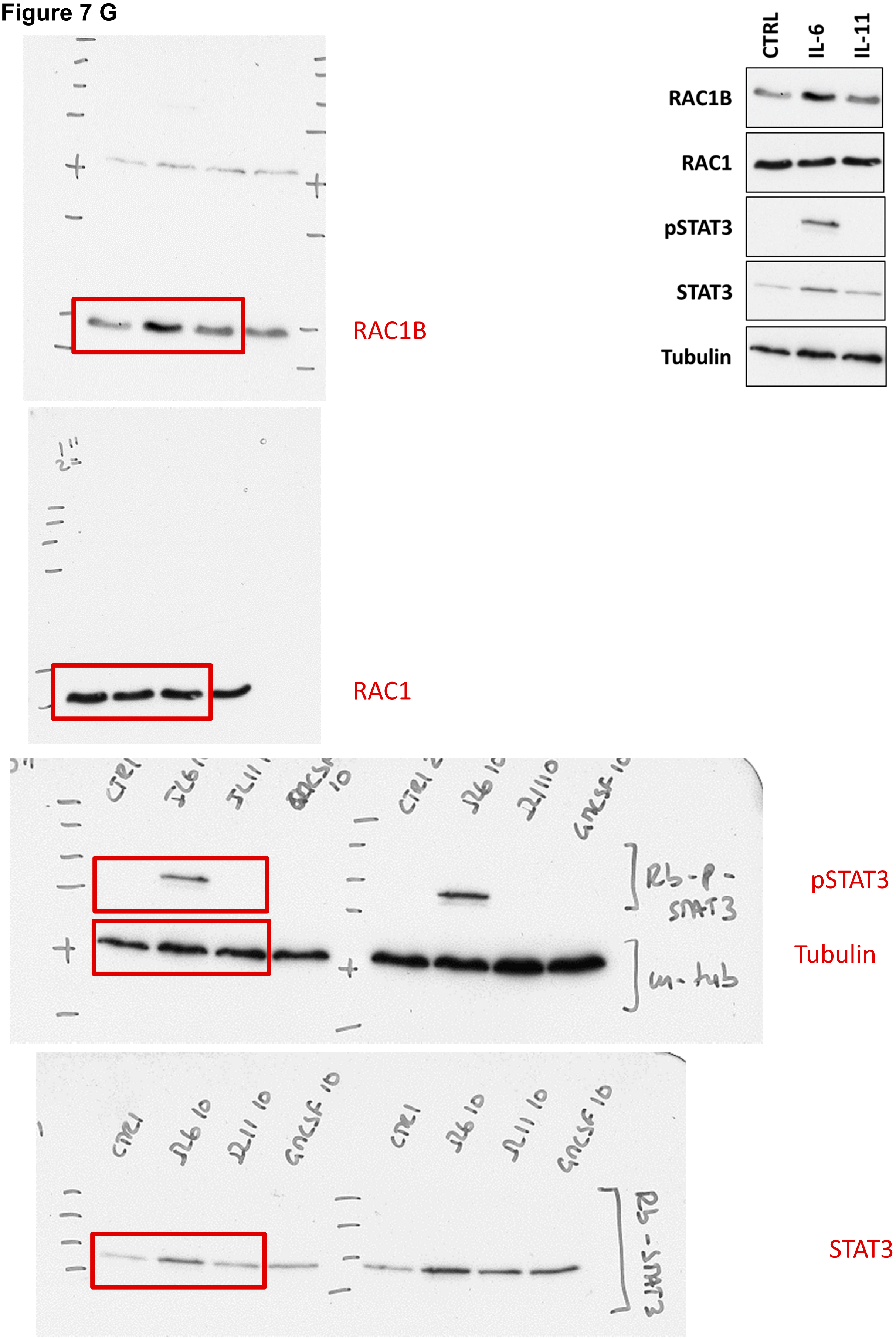
CTRL
IL-6
IL-11
RAC1B
RAC1
pSTAT3
STAT3
Tubulin
RAC1B
RAC1
pSTAT3
Tubulin
STAT3

Figure S1A

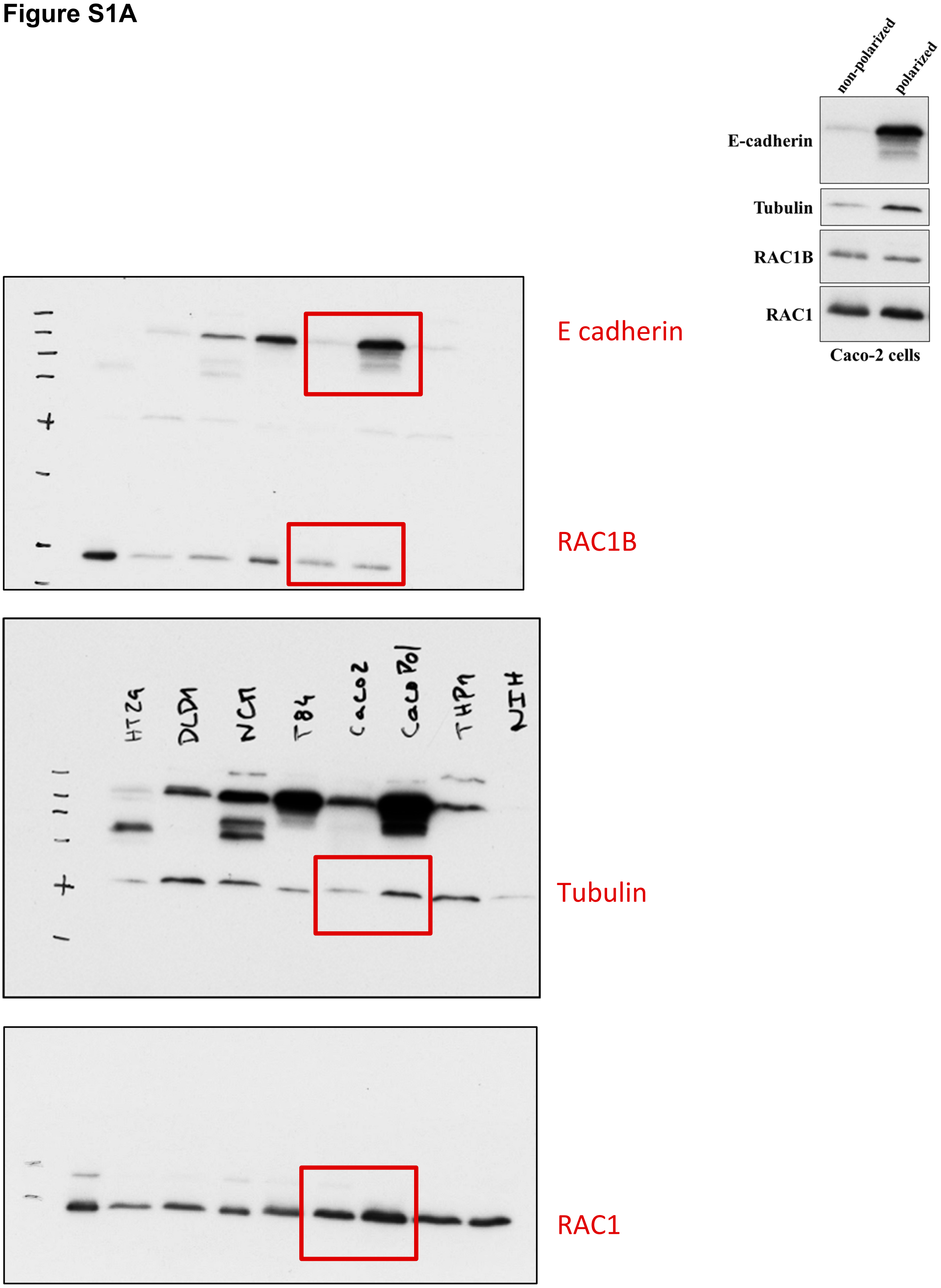
non-polarized
polarized
E-cadherin
Tubulin
RAC1B
RAC1
Caco-2 cells
E cadherin
RAC1B
HT29
DLD1
NCI
T84
Caco2
Caco Pol
NIH
Tubulin
RAC1

Figure S3A

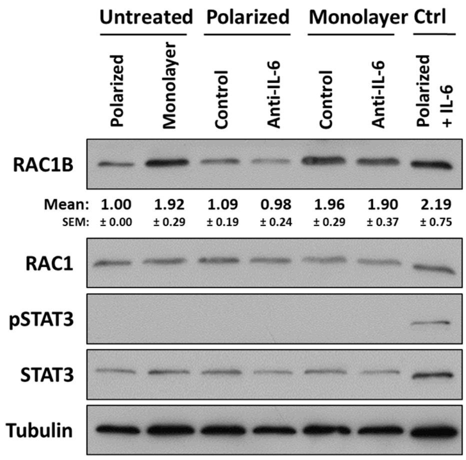

Untreated
Polarized
Monolayer Ctrl
Polarized
Monolayer
Control
Anti-IL-6
Control
Anti-IL-6
Polarized + IL-6
RAC1B
Mean: 1.00 1.92 1.09 0.98 1.96 1.90 2.19
SEM: ± 0.00 ± 0.29 ± 0.19 ± 0.24 ± 0.29 ± 0.37 ± 0.75
RAC1
pSTAT3
STAT3
Tubulin

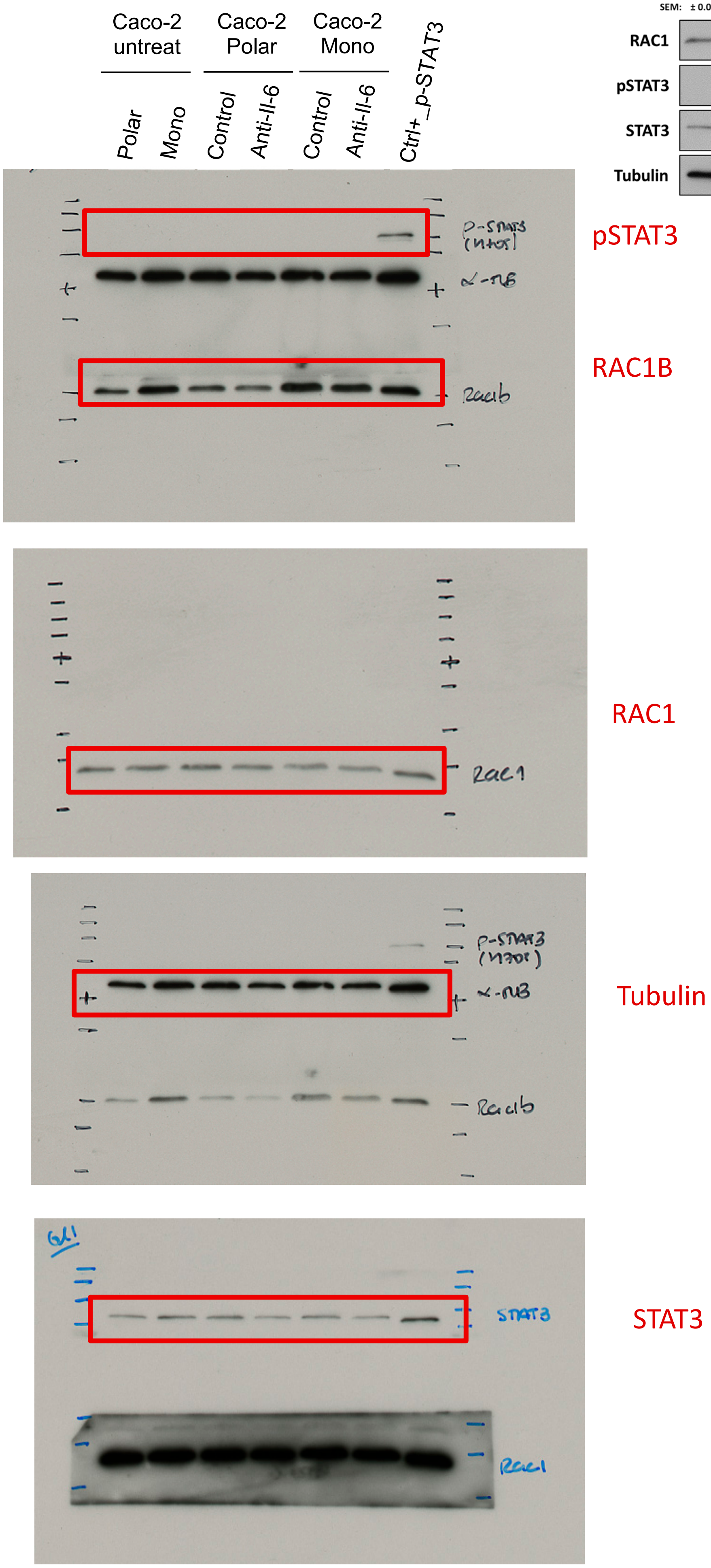

Caco-2 untreat
Caco-2 Polar
Caco-2 Mono
Polar
Mono
Control
Anti-Il-6
Control
Anti-Il-6
Ctrl+_p-STAT3
pSTAT3
RAC1B
RAC1
Tubulin
STAT3

Figure S3B

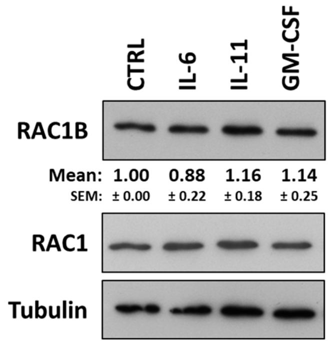

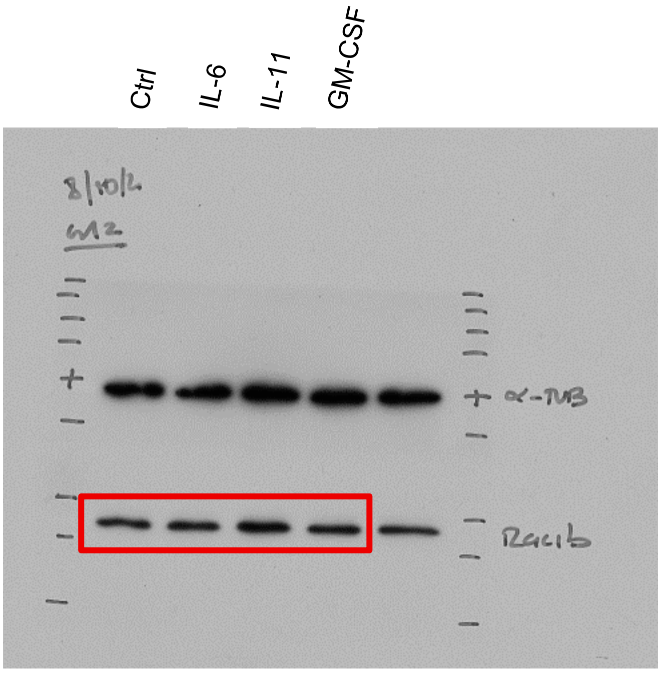

RAC1B

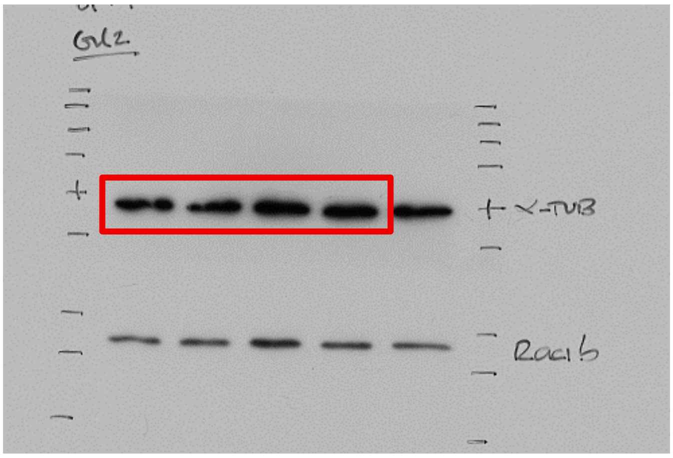

Tubulin

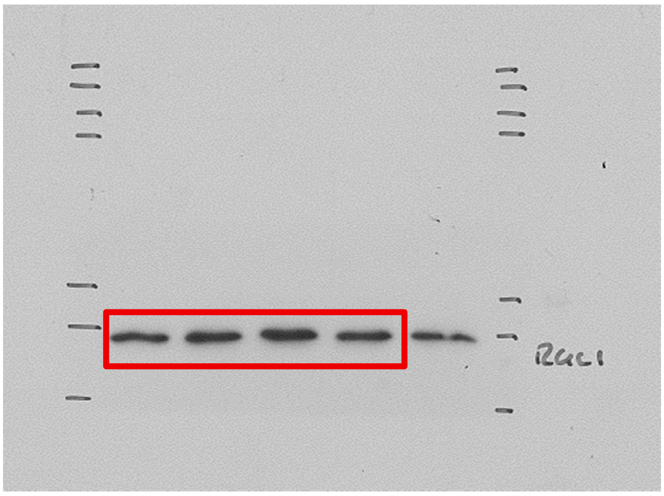

RAC1
