# Supplementary material for: Pro-Inflammatory Cytokines Trigger the Overexpression of Tumour-Related Splice Variant RAC1B in Polarized Colorectal Cells
Source: Cancers (Basel). 2022 Mar 9;14(6):1393. doi: 10.3390/cancers14061393 (PMC8946262; doi:10.3390/cancers14061393)
Supplement: Supplementary file 1 [file cancers-14-01393-s001.zip › Suppl File S2_supplementary Figures and Tables.pdf]

Supplementary File S2

Supplementary Figures and Tables

Pro-inflammatory cytokines trigger the overexpression of tumour-related splice variant RAC1B in polarized colorectal cells

Joana F. S. Pereira[1,2], Cláudia Bessa[1,2], Paulo Matos[1,2], Peter Jordan[1,2]

[1]Department of Human Genetics, National Health Institute Dr. Ricardo Jorge, Lisbon, Portugal; [2]BioISI – Biosystems & Integrative Sciences Institute, Faculty of Sciences, University of Lisbon, Lisbon, Portugal

Figure S1

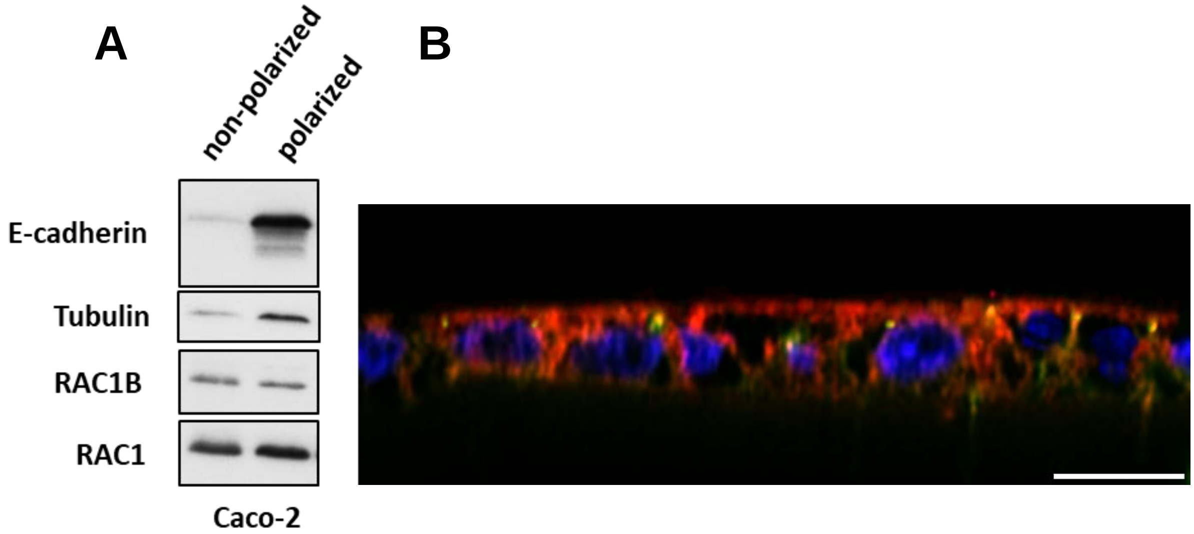

Supplementary Figure S1 – **E-cadherin and tight junctions in the polarized Caco-2 cells.** Cells were seeded either on plastic culture dishes and grown into a monolayer (non-polarized), or on filter inserts and grown for 12-14 days to allow for cell polarization (polarized). (**A**) Expression of the indicated proteins under both growth conditions. Proteins were analyzed by SDS-PAGE and WB and the indicated proteins detected in the whole-cell lysates. (**B**) Detection of tight-junction formation in polarized cells after 14 days of culture. Shown is the overlay of three confocal immunofluorescence images, which detected cell nuclei in blue (DAPI), the localization of protein ZO-1 in green, and the actin filament marker phalloidin in red.

Figure S2

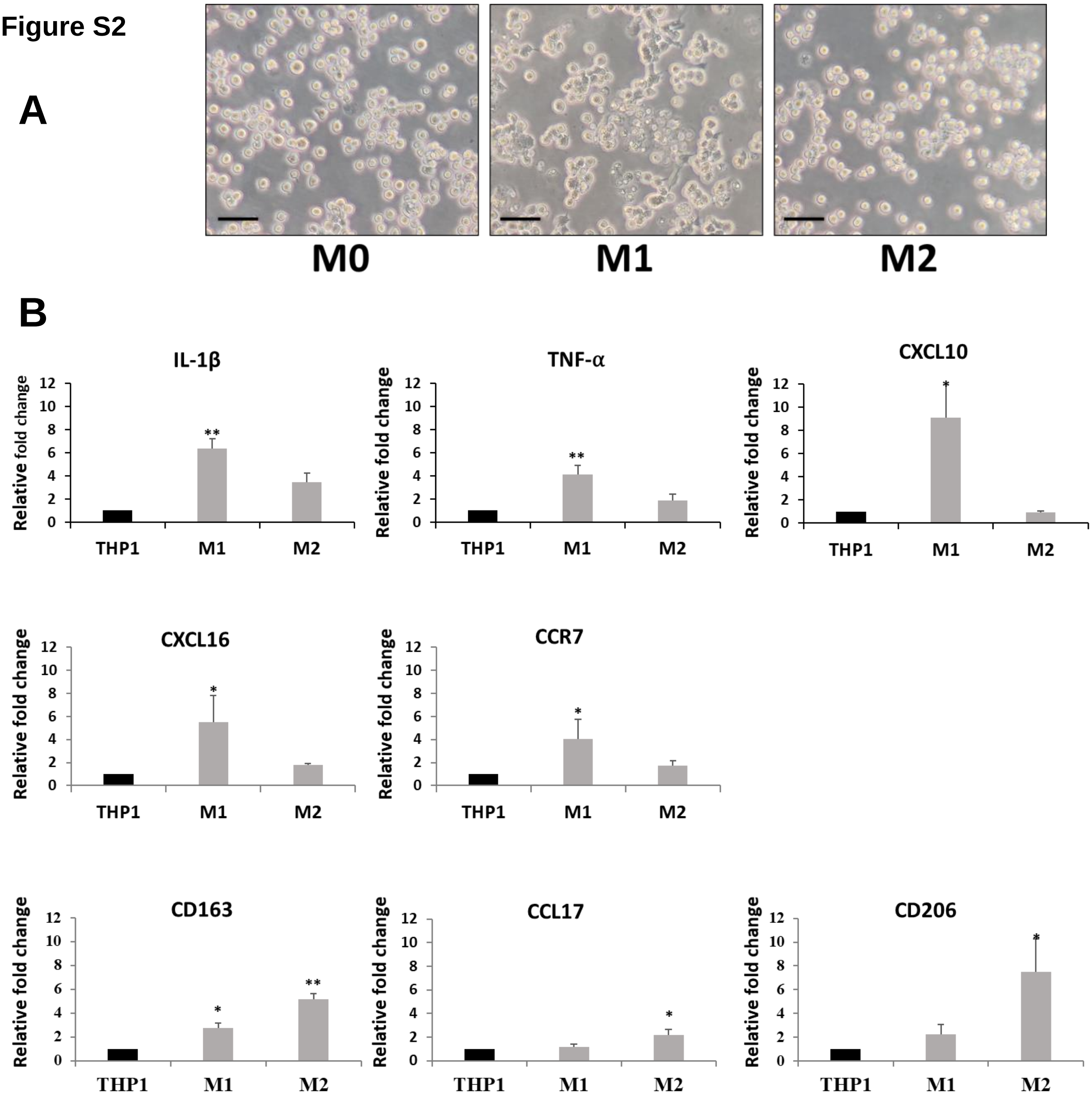

Supplementary Figure S2 – **Differentiation of THP-1 cells into macrophages.** (**A**) Photographs of THP-1 cells after 24 h of incubation with 50 ng/mL of PMA (designated M0 cells), of M0 cells after 24 h of incubation either with 10 ng/mL LPS and 10 ng/mL IFN-γ (designated M1 cells), or with 10 ng/mL IL-4 (designated M2 cells). Photographs were taken with a digital camera coupled to a bright-field microscope with a 40x magnification. Note the morphological alterations. (**B**) Expression of classical differentiation marker genes (M1: IL-1β, TNF-α, CXCL10, CXCL16 and CCR7; M2: CD163, CCL17 and CD206). Total RNA was isolated from each phenotype, reverse transcribed and amplified by PCR with GAPDH as an internal standard. The data represent the mean ± SEM of 3 independent experiments. Statistical analysis was carried out with a one-way ANOVA test followed by Tukey's post hoc test; * significantly different from the corresponding control (THP-1) with $p < 0.05$ or ** with $p < 0.01$.

Figure S3

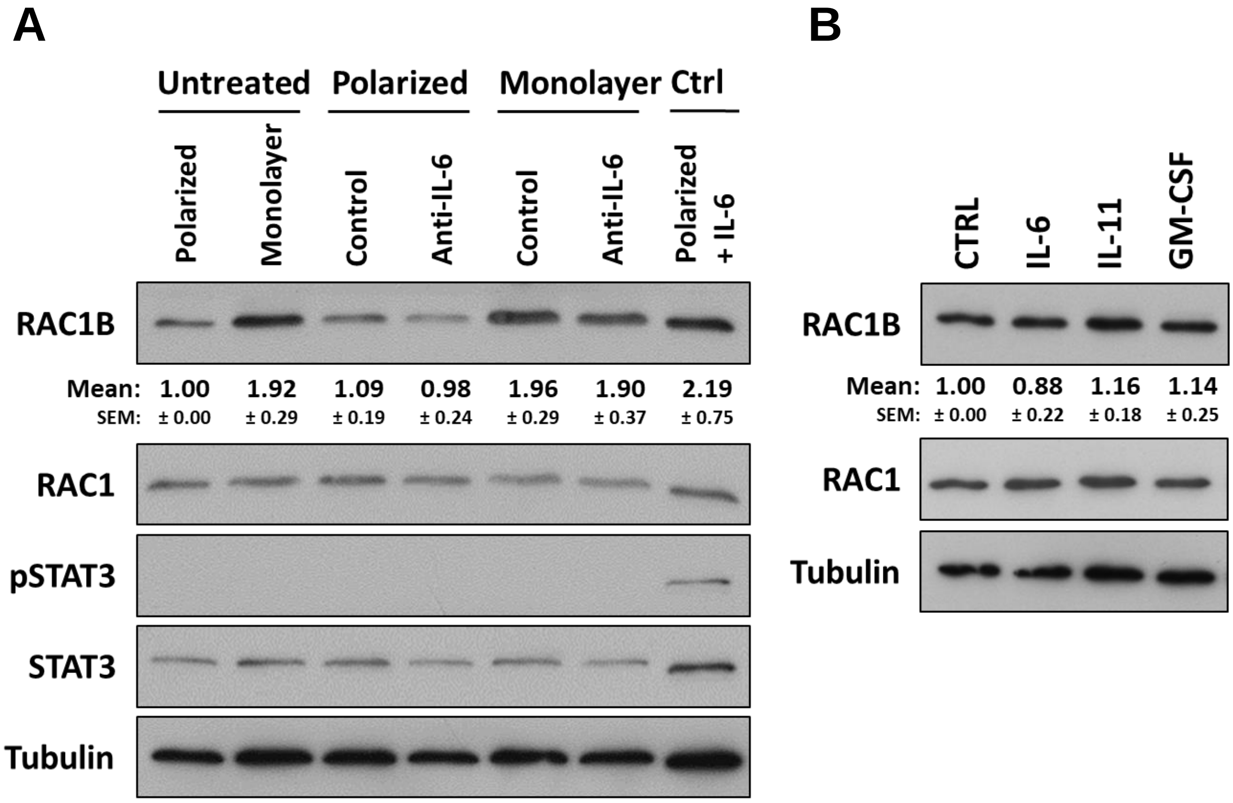

Supplementary Figure S3 – **Comparison of RAC1B levels after experimental manipulation of Interleukin (IL)-6 in polarized and non-polarized Caco-2 cells.**

(**A**) Colorectal Caco-2 cells were seeded on porous (1 µm) transwell PET filter inserts (24-well size, 6.4 mm diameter and 0.3 cm^2 area) in RPMI medium supplemented with 5% (v/v) FBS and then either grown for 10–15 days until they reached a TEER of 1000 to 1200 Ω (polarized), or left overnight for attachment (monolayer). Then the filters were added to 24-well plates containing 500 ng/mL of a neutralizing anti-IL-6 antibody in the basolateral medium. Cells were lysed after 48 h and proteins analysed by SDS-PAGE and WB of the whole-cell lysates. The control of the assay (Ctrl) corresponds to treatment of fully polarized Caco-2 cells with purified IL-6 in the basolateral medium. Band intensities were measured, and then normalized to tubulin levels, followed by normalization to total RAC1 levels (as both RAC1 and RAC1B are derived from the same pre-mRNA transcript). Intensity values are shown as fold-change of the RAC1B/RAC1 ratio relative to polarized, untreated cells and represent means ± SEM of 3 independent experiments. (**B**) Caco-2 cells were grown as a non-polarized monolayer, as described in (A), and then transferred to 24-well plates containing growth medium supplemented with 10 ng/mL of purified IL-6 or, as controls, 10 ng/mL of IL-11 or GM-CSF. Cells were lysed after 48 h and proteins analysed as in (A).

Note that RAC1B protein levels in non-polarized Caco-2 cells did not change after addition of neutralizing anti-IL-6 antibodies, nor did they correlate with increased STAT3 phosphorylation levels. Moreover, RAC1B levels in non-polarized cells did not respond to the addition of purified IL-6 (B). Thus, the difference between non-polarized and polarized Caco-2 cells observed in Fig. 1C does not seem to involve IL-6.

Table S1: Characteristics of the colorectal cell lines used

Cell line	Mutated genes	Origin	Microsatellite stability status	Phenotypic properties
Caco-2	APC Q1367*, CTNNB1 G245A, TP53 E204*, SMAD4 D351H	colon adenocarcinoma	MSS	differentiate into a polarized monolayer with functional characteristics of intestinal enterocytes;
T84	APC L1488fs*19, KRAS G13D, PI3KCA E542K, SMAD4 K340N	colon carcinoma, lung metastasis	MSS	differentiate into a polarized monolayer; mixed differentiation into both, chloride-secreting enterocytes and mucin-secreting goblet-like colonocytes
NCM460	cytogenetic alterations, lack p16INK4a expression	immortalized normal colonocytes	MSS	metabolic characteristics of normal mucosa, with low glycolytic rate and pentose phosphate synthesis but high tricarboxylic acid cycle activity
HT29	APC E853*, BRAF V600E, PIK3CA P449T TP53 R273H SMAD4 Q311*	colon adenocarcinoma	MSS	hypertriploid cell line, tumorigenic when injected into nude mice, forming well-differentiated adenocarcinoma consistent with primary grade I tumor, can differentiate into mature mucus-producing goblet cells
DLD-1	APC I1417L fs*2*, KRAS G13D, PI3KCA E545K; D549N TP53 S241F	colon adenocarcinoma, lung metastasis	MSI	tumorigenic when injected into nude mice, pseudodiploid cell line, can undergo EMT

Adapted from: Pereira JFS, Awatade NT, Loureiro CA, Matos P, Amaral MD, Jordan P (2016). The third dimension: new developments in cell culture models for colorectal research. Cell. Mol. Life Sci. 73, 3971–3989. https://doi.org/10.1007/s00018-016-2258-2

Table S2: Primers used for RT-PCR amplification reactions

(	Transcript	Primer sequences		Annealing temperature
**M1 markers***	IL-1β	F: 5’ CCACAGACCTTCCAGGAGAATG	R: 5’ GTGCAGTTCAGTGATCGTACAGG	66ºC
	TNF-α	F: 5’ CTCTTCTGCCTGCTGCACTTTG	R: 5’ ATGGGCTACAGGCTTGTCACTC	68ºC
	CXCL10	F: 5’ GAAAGCAGTTAGCAAGGAAAGGTC	R: 5’ ATGTAGGGAAGTGATGGGAGAGG	66ºC
	CCR7	F: 5’ TGGTGGTGGCTCTCCTTGTC	R: 5’ TGTGGTGTTGTCTCCGATGTAATC	66ºC
	CXCL16	F: 5’ ACTACACGAGGTTCCAGCTCC	R: 5’ CTTTGTCCGAGGACAGTGATC	64˚C
**M2 markers***	CD163	F: 5’ GTCGCTCATCCCGTCAGTCATC	R: 5’ GCCGCTGTCTCTGTCTTCGC	68ºC
	CD206	F: 5’ TACCCCTGCTCCTGGTTTTT	R: 5’ CAGCGCTTGTGATCTTCATT	68ºC
	CCL17	F: 5’ CGGGACTACCTGGGACCTC	R: 5’ CCTCACTGTGGCTCTTCTTCG	66ºC
	CD36	F: 5’ GAGAACTGTTATGGGGCTAT	R: 5’ TTCAACTGGAGAGGCAAAGG	60ºC
**Control**	GAPDH	F: 5’ GTCTCCTCTGACTTCAACAGCG	R: 5’ ACCACCCTGTTGCTGTAGCCAA	66ºC
**Caco-2****	IL-6	F: 5’ ATGAACTCCTTCTCCACAAGCC	R: 5’ GAAGAGCCCTCAGGCTGGACTG	64ºC
**RAC1**	RAC1/-1B	F: 5’ CTTTGACAATTATTCTGCCAATG	R: 5’ CGGACATTTTCAAATGATGCAGG	60ºC
**qPCR**	RAC1B	F: 5’ GGGCAAAGACAAGCCGATTG	R: 5’ CGGACATTTTCAAATGATGCAGG	60ºC
	RAC1 total	F: 5’ CCTGCATCATTTGAAAATGTCCG	R: 5’ CCCACTAGGATGATGGGAGTGT	60ºC

(F= forward, R= reverse);* marker genes as described in:

Chanput W, Mes JJ, Savelkoul HFJ, Wichers HJ (2013). Characterization of polarized THP-1 macrophages and polarizing ability of LPS and food compounds. Food Funct 4: 266–276. doi:10.1039/C2FO30156C

Genin M, Clement F, Fattaccioli A, Raes M, Michiels C (2015). M1 and M2 macrophages derived from THP-1 cells differentially modulate the response of cancer cells to etoposide. BMC Cancer 15: 577. doi:10.1186/s12885-015-1546-9

Li H, Sun S, Lei Q, Lei P, Cai X, Wan C, Shen G (2018). M1-Polarized Macrophages Promote Self-Renewing Phenotype of Hepatic Progenitor Cells with Jagged1-Notch Signalling Involved: Relevance in Primary Sclerosing Cholangitis. J. Immunol. Res. 2018: e4807145. doi:10.1155/2018/4807145

Littlefield MJ, Teboul I, Voloshyna I, Reiss AB (2014). Polarization of Human THP-1 Macrophages: Link between Adenosine Receptors, Inflammation and Lipid Accumulation. Int J Immunol Immunother. 1: 001. doi: 10.23937/2378-3672/1410001

Wheeler KC, Jena MK, Pradhan BS, Nayak N, Das S, Hsu C-D, et al. (2018). VEGF may contribute to macrophage recruitment and M2 polarization in the decidua. PLoS ONE 13(1): e0191040. doi: 10.1371/journal.pone.0191040

** as described in:

Parikh AA, Salzman AL, Kane CD, Fischer E, Hasselgren PO (1997). IL-6 production in human intestinal epithelial cells following stimulation with IL-1 beta is associated with activation of the transcription factor NF-kappa B. J Surg Res 69(1):139-44. doi: 10.1006/jsre.1997.5061
